# Evaluation of an electron Monte Carlo dose calculation algorithm for treatment planning

**DOI:** 10.1120/jacmp.v16i3.4636

**Published:** 2015-05-08

**Authors:** Eve Chamberland, Luc Beaulieu, Bernard Lachance

**Affiliations:** ^1^ Département de physique de génie physique et d'optique, et Centre de recherche en cancérologie de l'Université Laval, Université Laval Québec City QC Canada; ^2^ Département de Radio‐Oncologie et Centre de recherche du CHU de Québec CHU de Québec Québec City QC Canada

**Keywords:** electron beams, Monte Carlo, treatment planning system, dose calculation

## Abstract

The purpose of this study is to evaluate the accuracy of the electron Monte Carlo (eMC) dose calculation algorithm included in a commercial treatment planning system and compare its performance against an electron pencil beam algorithm. Several tests were performed to explore the system's behavior in simple geometries and in configurations encountered in clinical practice. The first series of tests were executed in a homogeneous water phantom, where experimental measurements and eMC‐calculated dose distributions were compared for various combinations of energy and applicator. More specifically, we compared beam profiles and depth‐dose curves at different source‐to‐surface distances (SSDs) and gantry angles, by using dose difference and distance to agreement. Also, we compared output factors, we studied the effects of algorithm input parameters, which are the random number generator seed, as well as the calculation grid size, and we performed a calculation time evaluation. Three different inhomogeneous solid phantoms were built, using high‐ and low‐density materials inserts, to clinically simulate relevant heterogeneity conditions: a small air cylinder within a homogeneous phantom, a lung phantom, and a chest wall phantom. We also used an anthropomorphic phantom to perform comparison of eMC calculations to measurements. Finally, we proceeded with an evaluation of the eMC algorithm on a clinical case of nose cancer. In all mentioned cases, measurements, carried out by means of XV‐2 films, radiographic films or EBT2 Gafchromic films. were used to compare eMC calculations with dose distributions obtained from an electron pencil beam algorithm. eMC calculations in the water phantom were accurate. Discrepancies for depth‐dose curves and beam profiles were under 2.5% and 2 mm. Dose calculations with eMC for the small air cylinder and the lung phantom agreed within 2% and 4%, respectively. eMC calculations for the chest wall phantom and the anthropomorphic phantom also showed a positive agreement with the measurements. The retrospective dosimetric comparison of a clinical case, which presented scatter perturbations by air cavities, showed a difference in dose of up to 20% between pencil beam and eMC algorithms. When comparing to the pencil beam algorithm, eMC calculations are definitely more accurate at predicting large dose perturbations due to inhomogeneities.

PACS numbers: 87.55.de, 87.55.kd

## I. INTRODUCTION

Monte Carlo (MC) techniques are widely used in radiotherapy to simulate the propagation of photons and charged particles through matter.^1^, ^2^, ^3^ MC algorithms are considered to be the ultimate treatment planning dose calculation tool. No other algorithm can accurately calculate the dose perturbations of electron beams in complex situations, such as bones and air cavities. Millions of simulated particles (histories) are required for radiation therapy treatment planning to statistically obtain accurate dose distributions.^(4)^ These simulations are still much too expensive in calculation time for routine treatment planning unless time‐saving approximations are used. The electron Monte Carlo (eMC) dose calculation algorithm included in the Eclipse treatment planning system (Varian, Palo Alto, CA) is a fast implementation of the Monte Carlo method used for computation of dose, from high‐energy electron beam. It is based on standard EGS Monte Carlo methods^(5)^ and reduces the processing time by introducing simplifications to the calculation algorithm. The system's behavior of this commercial product has previously been studied,^6^, ^7^, ^8^ and ionization chamber dosimetry, film dosimetry, and Monte Carlo simulations were used to validate dose distributions with the eMC algorithm. It has been shown that this product is clinically acceptable, even for very complex 3D‐type inhomogeneities. Also, 6 MeV beams are not modeled as accurately as other beam energies. Moreover, the grid spacing should not be larger than approximately one‐tenth of the distal falloff distance of the electron depth‐dose curve (depth from 80% to 20% of the maximum dose). We are presenting our evaluation of the eMC algorithm employed by Eclipse. The study compares eMC calculations made on different geometries and real patient cases, film measurements, and calculations performed with an electron pencil beam algorithm. A calculation time evaluation was also performed to validate the clinical viability of this commercial product, especially since the introduction of the Distributed Calculation Framework that permits parallelized dose calculation.

## II. MATERIALS AND METHODS

The first step was to configure the eMC algorithm with measured beam data, such as depth‐dose curves and beam profiles in water. Then, we calculated plans on test geometries and real patient cases to explore the system's behavior in configurations met in clinical practice. The first test was performed in a water phantom, where measured and eMC‐calculated dose distributions were compared for various combinations of energy and applicator. Other tests were performed with three different inhomogeneous solid phantoms built by using high‐ and low‐density material inserts. The first phantom is an acrylic slab with a small cylindrical shaped hole in the center and the slab is imbedded in plastic water diagnostic therapy materials (PWDT, CIRS, Norfolk, VA). The second phantom simulates lung inhomogeneity and consists of cork slabs also imbedded in PWDT. The last test is a chest wall phantom, which contains Teflon bars (PolyAlto, Quebec City, QC, Canada) simulating the ribs and cork slabs for the lungs. Finally, the evaluation of the eMC algorithm on a clinical case of nose cancer was conducted. For all those tests, we used a statistical accuracy of 1% and 2% and a calculation grid size of 1×1,1.5×1.5 and $2\times 2\;{\text{mm}}^{2}$. In all cases, the measurements, carried out by means of radiographic or radiochromic films, were used to compare eMC calculations with dose distributions obtained from Pinnacle^3^ (Philips Medical Systems, Madison, WI) pencil beam algorithm. In this study, the criteria of acceptability set by Van Dyk et al.^(9)^ are used.

### A. The electron beam calculation algorithm

#### A.1 Description

The eMC algorithm consists of two models;^(10)^ 1) a transport model based on the Macro Monte Carlo developed by Neuenschwander et al.,^(5)^ which simulates the transport of electrons by calculating the dose deposition on each point, and 2) an Initial Phase Space model, which describes electrons and photons emerging from the treatment linear accelerator's^(11)^ head.

The Macro Monte Carlo method reduces the calculation time by simplifying the calculation algorithm. Its process is separated into three parts; local simulation, geometric preprocessing, and global simulation. The local simulation is a precomputation step that simulates the transport of monoenergetic electrons through macroscopic spherical volume elements, called kugels. Those precalculations are performed for different sphere radius (0.5, 1.0, 1.5, 2.0, and 3.0 mm), for five different materials (air: $0.001205\;g/{\text{cm}}^{3}$, lung phantom LN4: $0.3\;g/{\text{cm}}^{3}$, water: $1\;g/{\text{cm}}^{3}$, Lucite: $1.19\;g/{\text{cm}}^{3}$, and solid bone phantom SB3: $1.84\;g/{\text{cm}}^{3}$), and for 30 incident electron energies (0.2 to 25 MeV). It generates a list of probability distribution functions (PDF) which describes the exit position, the direction, and the energy of the electron emerging from the sphere. The geometric preprocessing step determines the sphere size and its mean density at every CT image position. Depending on the proximity of the interface, the sphere size will decrease when nearing the interface. Finally, the global simulation transports electrons through the patient CT with macroscopic steps, in order to determine the electron beam's dose deposition.

All the detailed information on the nature of every particle emerging from the head of the linear accelerator represents the phase space. The Initial Phase Space model used in the eMC algorithm is represented by a four‐source model: 1) primary electrons and photons originating in the scattering foils, 2) a virtual source of scattered electrons and photons, 3) electrons scattered from the edges of the electron collimating device, and 4) a source of photons generated in the applicator. The probability distributions of position, energy, and direction of electrons and photons are given at the phase‐space plane under the electron applicator. The Initial Phase Space is adjusted to match our linear accelerator measurements. Its configuration requires multiple measurements:

1. Open beam measurements for all energies:
  - Percent depth dose in water at a source‐to‐surface distance of 100 cm.
  - Absolute dose (cGy/MU) in water at depth of maximum dose.
  - Profile in air at 95 cm from the source.
2. Applicator measurements for all energies
  - Percent depth dose in water at a source‐to‐surface distance of 100 cm.
  - Absolute dose (cGy/MU) in water at depth of maximum dose.

#### A.2 Calculation parameters

The treatment planner has different calculations parameters to select: the statistical accuracy (1% to 8%), the calculation grid size $\left(1\times 1\;\text{to}\;5\times 5\;{\text{mm}}^{2}\right)$, the smoothing method (3D Gaussian or 2D median), the smoothing level (low, medium, high), and the seed number used for the random generator. The statistical accuracy refers to the mean statistical error in dose for all voxels receiving more than 50% of the maximum dose value. The algorithm will continue the simulation until the stated mean statistical accuracy is achieved. The calculation grid size is used to control the grid size X and Y DICOM axes. The grid size in the third dimension, the Z DICOM axis, is the same as the CT slice spacing. The calculation grid size also defines the resolution used in the geometric preprocessing phase. The seed number (or random number generator seed) initiates the state of the random number generator. Various seed numbers generate slightly different calculations, but they all remain within the defined level of accuracy. Both the influence of the seed number and the calculation grid size were evaluated in a water phantom.

##### A.2.1 Seed number

It is required to evaluate the statistical dispersion and the systematic error of a large number of calculations for various seed numbers. We suppose that the statistical character of the Monte Carlo calculation has a normal (Gaussian) distribution. The employed method consists on performing, in a virtual water phantom, ten calculations with different seed numbers from 1 to 3100000000 (49000000, 49100000, 49300000, 50300000, 50500000, 50700000, 50900000, 51000000, 52000000, and 401000000). All other calculation parameters are kept constant. For each calculation, the depth dose curve is exported. Then, the mean and the standard deviation of every curve can be calculated and compared with the experimental measurement. A 95% confidence interval (±2 σ) is considered for the statistical dispersion evaluation.

This study was performed at 18 MeV, for a source‐to‐surface distance of 100 cm and for two different field sizes $\left(6\times 6\;{\text{cm}}^{2},4\times 4\;{\text{cm}}^{2}\right)$. Calculations are done with a 2% statistical accuracy and a grid size of $2\times 2\;{\text{mm}}^{2}$. The evaluation methodology depends on the region of the depth‐dose curve. The majority of deviations for a depth‐dose curve are given in term of % dose difference, except for large dose gradient regions, where deviations are expressed in term of distance to agreement (mm) (ICRU 42^(12)^, Van Dyk et al.^(9)^). The analysis regions of depth dose curves are:

- dmax‐d80%: evaluation in term of % dose difference (fixed depth)
- d80%‐d20% (large dose gradient region): evaluation in term of distance to agreement (mm) (fixed dose)
- d20%‐end: evaluation in term of % dose difference (fixed depth)

where ‘dmax’ is the depth of the maximum dose, the ‘dx%’ is the depth at x% of the depth‐dose curve, and ‘end’ represents the end of the measured depth‐dose curve.

##### A.2.2 Calculation grid size

The method employed is to compare depth‐dose curves for different calculation grid sizes. This study was performed for different energies and field sizes. Only the first two grid sizes were evaluated: 1×1 and $2\times 2\;{\text{mm}}^{2}$. These results will be also compared with those of Popple et al.^(6)^ They showed that the grid size should not be larger than one‐tenth of the dose falloff (depth from 80% to 20% of the maximum dose) of the electron depth‐dose curve. To be more specific, the grid size must be $1\times 1\;{\text{mm}}^{2}$ at 6 MeV and 9 MeV, $1.5\times 1.5\;{\text{mm}}^{2}$ at 12 MeV, $2\times 2\;{\text{mm}}^{2}$ at 15 MeV, and $2.5\times 2.5\;{\text{mm}}^{2}$ at 18 MeV.

#### A.3 Algorithm performance

##### A.3.1 Output factors

Electron beam are calibrated to give 1cGy per monitor unit (MU) at the depth of maximum dose. MU calculation is an important verification for every planning system validation. This is done by comparing calculated and measured output factors (OF) in a water phantom.

(1) $$OF=\frac{D_{cutout}(C,A,SSD)}{D_{ref}(c=15\times 15\;cm^{2},A=15\times 15\;cm^{2},\;SSD=100\;cm)}$$

where $D_{cutout}(C,A,SSD)$ is the dose at dmax for a cutout field size *c*, in an applicator size *A* and a source‐to‐surface *SSD*, per 100 MU delivered; $D_{ref}$ is the dose at the reference point $\left(15\times 15\;{\text{cm}}^{2}\right)$ per 100 MU delivered.

The study is performed for three energies (6, 12, and 18 MeV) and for different field sizes which are 3×3,4×4,6×6,8×8,10×10,15×15, and $20\times 20\;{\text{cm}}^{2}$. All calculations are done with a 1% statistical accuracy and a grid size of $1\times 1\;{\text{mm}}^{2}$. Calculated outputs are averaged over a small region of interest in order to eliminate statistical errors due to the stochastic nature of Monte Carlo.

##### A.3.2 Calculation time

A calculation time evaluation is required to see how fast the eMC algorithm is and also to determine if this product is clinically viable. Since the 8.1 version of Eclipse, the introduction of the Distributed Calculation Framework (DCF) allows parallelized dose calculation. The DCF increases the dose calculation speed because calculation tasks can be performed on multiple workstations and multiple processors simultaneously.

In this study, all calculations were initially performed with a 3.2 GHZ Intel Xeon Processor (Intel Corporation, Santa Clara, CA) with 2.8 GB of RAM without DCF. Furthermore, calculation times are compared when running on a 2.4 GHZ Intel Dual Quad core Xeon Processor with 24 GB of RAM and by using the DCF. Calculations were performed at three energies (6, 12, and 18 MeV) with two field sizes (6×6 and $20\times 20\;{\text{cm}}^{2}$). Different grid sizes ($1.5\times 1.5\;{\text{mm}}^{2},2\times 2\;{\text{mm}}^{2},2.5\times 2.5\;{\text{mm}}^{2}$, and $5\times 5\;{\text{mm}}^{2}$) and statistical accuracies (1%, 2%, 3%, and 5%) were used.

### B. Film dosimetry

#### B.1 Radiographic film dosimetry

All beam profiles in solid phantoms were measured with Kodak XV‐2 radiographic films (Eastman Kodak Company, Rochester, NY). This film is linear for doses up to 50 cGy and it saturates at approximately 100 cGy. A calibration curve is essential to characterize optical densities for doses in the nonlinear region. This is performed by measuring 12 individual films at the same depth of reference (at depth of maximum dose: 1 cGy/MU), from 0 to 60 cGy. Films are then scanned using a VIDAR VXR‐16 dosimetry pro scanner (VIDAR Systems, Herndon, VA). The optical density to dose calibration curve is obtained by performing a polynomial fit to the measurements.

#### B.2 Radiochromic film dosimetry

All depth‐dose curves in solid phantoms were measured with Gafchromic films (Gafchromic EBT2, International Specialty Products, Inc., Wayne, NJ). Radiographic XV‐2 film is not a good choice for those measures because it is subject to surface artifact when the film border is not perfectly aligned with the surface itself. Also, some artifacts may arise if there is air inside the film envelope.

EBT2 films are insensitive to visible light, they are closely equivalent to water, and they can be used for doses up to 800 cGy. Before the scan, it is recommended to wait for at least 24 hrs, for its optical density stabilization.

The calibration of those EBT2 films is performed by doing several expositions at a reference depth, for doses ranging from 10 to 500 cGy. Every film is then scanned five times with an Epson 10000XL flatbed scanner (US Epson, Long Beach, CA) at 75 dpi and 48 bpp RGB format. All images are averaged and the red component of the resultant image is extracted. The film response is increased with a measure in the red canal because the absorption peak of the active component of the film after its exposition is approximately 636 nm (red). And then, finally, a uniformity correction of the scanner is applied. The correlation between the optical density and the dose is obtained by doing an independent measurement with a Farmer ionization chamber (Exradin A12; Standard Imaging Inc., Middleton, WI) at the same depth.

### C. Ionization chamber measurements

All beam profiles and depth‐dose curves in water required for the eMC algorithm configuration were measured with an ionization chamber IC10 (Wellhofer; Scanditronix–Wellhofer, Nuremburg, Germany; 6 mm of diameter, cavity volume 0.13 cm^3^).

The estimated uncertainties of measurements were 1%‐2%.

### D. Phantoms

The eMC algorithm validation required several measurements in different test geometries — homogeneous and inhomogeneous phantoms simulating high‐ and low‐density materials. All measurements are performed with a Clinac iX accelerator (Varian Medical Systems). All the beam energies (6, 9, 12, 15, and 18 MeV) are commissioned for this machine. In this study, experimental measurements are compared with pencil beam and eMC dose distributions.

#### D.1 Water phantom

The first geometry used was a simple water phantom (Blue phantom, Wellhofer, IBA dosimetry, Schwarzenbruck, Germany). Measured and eMC‐calculated dose distributions, such as beam profiles and depth‐dose curves, are compared for different combinations of energy (6, 12, and 18 MeV) and applicator $\left(6\times 6,10\times 10,20\times 20\;{\text{cm}}^{2}\right)$, for the SSD of 100 cm. Oblique and extended SSD incident beams are also evaluated (a gantry angle of 20° and an SSD of 110 cm). The depth‐dose curves evaluation is separated into four groups: from 0.5 cm of depth to dmax, from dmax to depth of 80%, from depth of 80% to depth of 20%, and from depth of 20% to the end. Deviations are in majority given in percent, except for the gradient regions (80%‐20%), where deviation is expressed in terms of distance (mm). The beam profiles evaluation compares two parameters — the penumbra and the maximum dose.

#### D.2 Air cylinder

The second phantom is an acrylic slab $\left(1.06\;g/{\text{cm}}^{3}\right)$ of $29\times 29\times 2.6\;{\text{cm}}^{3}$ with a small cylindrical shaped hole in the center. The hole is 0.6 cm under the surface and it has a diameter of 1.278 cm and a height of 2 cm. Under the acrylic slab, 12 cm of plastic water slabs (PWDT, CIRS) is used. Beam profiles are measured with XV‐2 films at 12 MeV with a $10\times 10{\text{cm}}^{2}$ field. Films are inserted at three different depths (2.7 cm, 4.2 cm, and 6.3 cm) perpendicularly to the beam.

#### D.3 Lung phantom

The first version of this phantom consists of cork slabs imbedded in plastic water (PWDT, CIRS). Four Cork slabs with mass density of $0.2\;g/{\text{cm}}^{3}$ and overall dimension of $25\times 25\times 6.3\;{\text{cm}}^{3}$ are used to simulate lung. We used a 2 cm of plastic water material above, as well as 7 cm of plastic water for backscatter. This phantom is irradiated at 12 MeV and 18 MeV, with a $10\times 10\;{\text{cm}}^{2}$ field. For each measurement, XV‐2 films are inserted at three different depths perpendicularly to the beam in order to measure beam profiles. The statistical accuracy was set at 1% and the grid size to $1.5\times 1.5\;{\text{mm}}^{2}$. No normalization was performed.

A second version of a lung phantom was built and the major difference being the replacement of plastic water by real water. The cork has a dimension of $8\times 25\times 25\;{\text{cm}}^{3}$ and is submerged 2 cm below the water. This lung phantom is used to measure a depth‐dose curve at 12 MeV, with a $10\times 10\;{\text{cm}}^{2}$ field. In this geometry, the 2 cm plastic water at the surface was replaced with real water, in order to avoid surface artifacts on the film, due to the rounded corners of the plastic water slabs. Measurement was done with a radiochromic EBT2 film because it can be used in water.

#### D.4 Chest wall phantom

**Figure 1:**
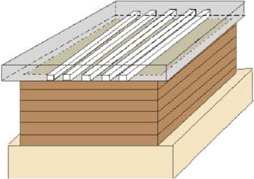
Phantom simulating a chest wall.

The chest wall phantom, shown in Fig. 1, is a complex geometry because it contains high‐ and low‐density heterogeneities. At the surface, there is a slab of polystyrene $30\times 30\times 2.6\;{\text{cm}}^{3}$. At a depth of 1.33 cm, there are five rectangular Teflon bars simulating ribs. Those bars of $1.27\times 30\times 1.27\;{\text{cm}}^{3}$ and of $2.16\;g/{\text{cm}}^{3}$ of mass density are each separated by 1.5 cm. Under those bars, cork slabs with a mass density of $0.2\;g/{\text{cm}}^{3}$ and an overall dimension of $20\times 20\times 6\;{\text{cm}}^{3}$ are used to simulate the lung. We used plastic water below the cork for backscatter. Beam profiles are measured with XV‐2 films, which are inserted at two depths perpendicularly to the beam. Measurements are performed at 12 MeV and 18 MeV, with a $20\times 20\;{\text{cm}}^{2}$ field. For eMC settings, the accuracy was set at 2% and the grid size at $1\times 1\;{\text{mm}}^{2}$. No normalization was performed.

#### D.5 Anthropomorphic phantom

**Figure 2:**
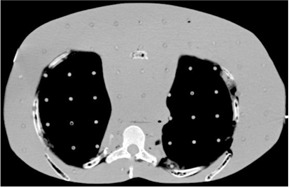
CT slice of the thorax part of RANDO.

The last geometry is an anthropomorphic phantom, RANDO (Alderson Research Labs, Stanford, CA), which is patient‐equivalent. It is composed of soft tissues composites, which are molded into a human skeleton. It includes materials simulating lung tissues and air cavities. Figure 2 shows a CT slice of RANDO's thorax part which is under study. Dose distributions are measured with a radiochromic EBT2 film, inserted inside RANDO's thorax part. This study is being done for an 18 MeV electron beam and for a $15\times 15\;{\text{cm}}^{2}$ applicator. For eMC settings, the accuracy was set at 2% and the grid size to $1.5\times 1.5\;{\text{mm}}^{2}$.

### D. Dose calculations

#### E.1 Eclipse eMC

All phantoms were scanned and exported into Eclipse in order to calculate dose distributions with the eMC algorithm (version 8.6) and to compare them with measurements. All calculations were smoothed by a 3D Gaussian filter of medium strength, in order to filter out any statistical noise in the final dose distribution. For all these tests, we used a statistical accuracy of 1% or 2% and a calculation grid size which respects Popple et al.^(6)^ criterion (1×1,1.5×1.5, or $2\times 2\;{\text{mm}}^{2}$) according to the desired energy.

#### E.2 Pinnacle

All phantom scans were also exported into Pinnacle^3^ (version 7.6; Philips Healthcare, Andover, MA), another commercial treatment planning system that uses the Hogstrom pencil beam algorithm (PBA).^(13)^ This particular one is largely used for calculating dose distributions when using electron beams in radiotherapy. In our clinic, a 3 mm grid size is generally used. For this study, the voxel size for all calculations was set at 2 mm.

### F. Clinical case data

Anatomic sites at low depths can be adequately treated with electron beams. They allow a precise dose deposit to superficial cancers by limiting the dose to underlying normal structures. A comparison between the eMC and the PBA is shown for one particular case of nose cancer. The patient in this study presents a basocellular carcinoma of the nose area and he had to have a total excision of his nose. There was a flat wax molded to his face in order to avoid irregular surfaces. A 60 Gy dose prescription in 30 fractions was prescribed for a 9 MeV electron beam. For both algorithms, 210 MU were delivered. For the eMC calculation, the statistical precision used was 1% and the calculation grid size was set at $1\times 1\;{\text{mm}}^{2}$.

## III. RESULTS

### A. eMC algorithm

#### A.1. Calculation parameters evaluation

##### A.1.1 Seed number

**Figure 3:**
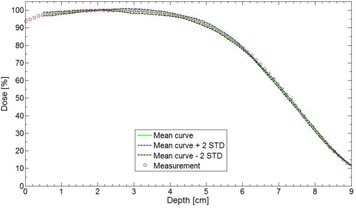
Seed number effect in water for a $6\times 6\;{\text{cm}}^{2}$ field size at 18 MeV and SSD=100 cm. Ten calculations with different seed numbers are averaged and a dispersion of ±2σ is also calculated. The measurement was done with an ionization chamber.

Figure 3 shows the evaluation of the effect of the seed number in water for a 18 MeV beam with a field size of $6\times 6\;{\text{cm}}^{2}$. The experimental curve is compared with the mean curve and the statistical dispersion for a total of ten calculations using different seed numbers. Table 1 shows the maximum systematic error and the maximum statistical dispersion for different regions of the depth dose curve of this 18 MeV beam. More specifically, the maximum systematic error is the maximum difference between a measurement and the mean curve, while the maximum statistical dispersion is the maximum difference between a measurement and the mean curve ±2σ. Table 2 presents another seed number evaluation for an 18 MeV beam with a field size of $4\times 4\;{\text{cm}}^{2}$.

**Table 1:** Analysis of systematic errors and statistical dispersions in water, for a field of $6\times 6\;{\text{cm}}^{2}$ at 18 MeV and SSD=100 cm. The statistical accuracy was 2% and the grid size was $2\times 2\;{\text{mm}}^{2}$.

*Curve Region*	*Maximum Systematic Error*	*Maximum Statistical Dispersion*
dmax – d80%	−0.3%	1.5%
d80% – d20%	−0.4 mm	−0.8 mm
d20% – end	0.2%	1.0%

**Table 2:** Analysis of systematic errors and statistical dispersions in water for a field of $4\times 4\;{\text{cm}}^{2}$ at 18 MeV and SSD=100 cm. The statistical accuracy was 2% and the grid size was $2\times 2\;{\text{mm}}^{2}$.

*Curve Region*	*Maximum Systematic Error*	*Maximum Statistical Dispersion*
dmax – d80%	0%	1.5%
d80% – d20%	0.4 mm	1.5 mm
d20% – end	0.4%	0.8%

In Fig. 3, the experimental curve (circles) is very close to the mean curve of the ten calculations (green). This shows that the systematic error is low. In Table 1, the systematic error is low and the maximum statistical dispersion is 1.5%, which is meeting the statistical accuracy parameter used (2%). In the gradient region (80%‐20%), the maximum dispersion is −0.8 mm, which is very acceptable because it is under the 2 mm grid resolution used. In Table 2, the results for the 18 MeV and a field size of $4\times 4\;{\text{cm}}^{2}$ are also very good for this experimental configuration. The systematic error is low and it is of opposite sign to the one observed with the $6\times 6\;{\text{cm}}^{2}$ field size. For this particular experimental condition, the accuracy of the calculation will be inside 1.5% and 1.5 mm 95% of the time, which again is meeting the defined level of accuracy (that is 2% and 2 mm).

##### A.1.2 Calculation grid size

**Figure 4:**
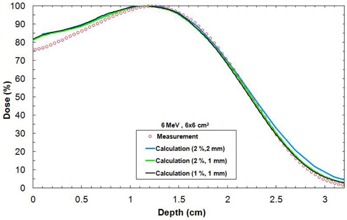
Depth‐dose curves in water for different resolution parameters for a $6\times 6\;{\text{cm}}^{2}$ field size at 6 MeV and SSD=100 cm. The measurement was done with an ionization chamber.

Figure 4 shows a comparison of measured and calculated depth dose curves with different grid sizes, in a water phantom at 6 MeV with a $6\times 6\;{\text{cm}}^{2}$ field size.

Using a statistical accuracy of 1% does not significantly improve the agreement with calculation when compared to a calculation using a 2% statistical accuracy. Using a $1\times 1\;{\text{mm}}^{2}$ grid size gives a better agreement with measurement when compared to a $2\times 2\;{\text{mm}}^{2}$ grid size. A 6 MeV beam should never be calculated with a $2\times 2\;{\text{mm}}^{2}$ grid size because there are discrepancies in the end of the depth‐dose curve. This study was also performed with other energies and the conclusions are similar to Popple et al.^(6)^ The grid size must be $1\times 1\;{\text{mm}}^{2}$ at 6 MeV and 9 MeV, $1.5\times 1.5\;{\text{mm}}^{2}$ at 12 MeV, $2\times 2\;{\text{mm}}^{2}$ at 15 MeV, and $2.5\times 2.5\;{\text{mm}}^{2}$ at 18 MeV.

#### A.2 Algorithm performance

##### A.2.1 Output factors

Table 3 shows comparison of eMC calculated and measured OF in a water phantom.

**Table 3:** Measured and eMC calculated output for different energies and field size in water at SSD=100 cm. eMC‐calculated outputs are averaged over a small region of interest in order to eliminate statistical errors. The error (±x) of these averaged outputs is given in the table. The symbol Δ (%) represents the % difference between the measured output and the eMC output. In the ‘Field’ column, $\text{Az}=\text{applicator of size z}\times {\text{z cm}}^{2}$ and $\text{Czxz}=\text{cutout of size z}\times {\text{z cm}}^{2}$.

	*6 MeV*			*12 MeV*			*18 MeV*		
*Field*	*Measure*	eMC±0.002	ΔΔ (%)	*Measure*	eMC±0.002	Δ (%)	*Measure*	eMC±0.003	Δ (%)
A6,C3×3	0.922	0.915	−0.7	0.933	0.925	−0.8	1.033	1.015	−1.7
A6,C4×4	0.966	0.963	−0.3	0.950	0.954	0.4	1.029	1.015	−1.4
A6,C6×6	0.968	0.965	−0.3	0.983	0.979	−0.4	1.022	1.014	−0.8
A15,C3×3	0.940	0.954	1.5	0.931	0.940	1.0	1.017	0.992	−2.4
A15,C6×6	1.026	1.002	−2.3	1.018	1.002	−1.6	1.036	1.004	−3.0
A15,C10×10	1.014	1.003	−1.1	1.017	1.000	−1.7	1.018	0.995	−2.2
A15,C15×15	1.000	0.990	−1.0	1.000	0.999	−0.1	1.000	0.987	−1.3
A20,C3×3	0.927	0.965	4.1	0.902	0.937	−3.9	0.982	0.974	−0.8
A20,C6×6	1.029	1.009	−1.9	0.997	0.986	−1.1	1.007	0.997	−1.0
A20,C8×8	1.030	1.012	−1.8	1.007	0.998	−0.9	1.008	0.990	−1.8
A20,C20×20	1.011	1.002	−0.9	0.987	0.980	−0.7	0.982	0.968	−1.4

The agreement is generally within 2% for cutouts being larger than $3\times 3\;{\text{cm}}^{2}$, except for one result (18 MeV, A15, $6\times 6\;{\text{cm}}^{2}$) which had a difference of 3%. For cutouts of $3\times 3\;{\text{cm}}^{2}$, the agreement is within 4%.

##### A.2.2 Calculation time

Table 4 shows the calculation times for different calculation parameters at a fixed energy and a fixed field size (6 MeV, $20\times 20\;{\text{cm}}^{2}$). Table 5 shows the calculation times for different energy and field sizes, while keeping statistical accuracy and grid sizes constant (2%, $2\times 2\;{\text{mm}}^{2}$). These two constants were only chosen to avoid long calculation times. In both 4, 5, calculations were performed with 3.2 GHZ Intel Xeon Processor with 2.8 GB of RAM, without the use of DCF. Table 6 shows a comparison of calculation times when running on a 2.4 GHZ Intel Dual Quad core Xeon Processor with 24 GB of RAM, with the use of parallel computing. In particular, calculation times with and without DCF are compared for different calculation parameters, randomly chosen. Energy and field sizes were kept fixed (6 MeV, $20\times 20\;{\text{cm}}^{2}$).

**Table 4:** Calculation times for different calculation parameters (6 MeV, $20\times 20\;{\text{cm}}^{2}$). The DCF is not used.

	*Statistical Accuracy*	*Grid Size*	*Calculation Time*
Fixed grid size	5%	$2\times 2\;{\text{mm}}^{2}$	2.60 min
	3%	$2\times 2\;{\text{mm}}^{2}$	7.65 min
	2%	$2\times 2\;{\text{mm}}^{2}$	16.95 min
	1%	$2\times 2\;{\text{mm}}^{2}$	68.20 min
Fixed statistical accuracy	2%	$2\times 2\;{\text{mm}}^{2}$	1.08 min
	2%	$2.5\times 2.5\;{\text{mm}}^{2}$	5.02 min
	2%	$2\times 2\;{\text{mm}}^{2}$	16.95 min
	2%	$1.5\times 1.5\;{\text{mm}}^{2}$	29.72 min

**Table 5:** Calculation times for various energies and field sizes, while statistical accuracy and calculation grid size are kept constant (2%, $2.5\times 2.5\;{\text{mm}}^{2}$). The DCF is not used.

*Energy*	*Field Size*	*Calculation Time*
6 MeV	$6\times 6\;{\text{cm}}^{2}$	1.78 min
	$20\times 20\;{\text{cm}}^{2}$	16.95 min
12 MeV	$6\times 6\;{\text{cm}}^{2}$	4.23 min
	$20\times 20\;{\text{cm}}^{2}$	28.25 min
18 MeV	$6\times 6\;{\text{cm}}^{2}$	7.00 min
	$20\times 20\;{\text{cm}}^{2}$	48.00 min

**Table 6:** Comparison of calculation times for different calculation parameters (statistical accuracy (%) and calculation grid size (mm)), with and without DCF. For this study, the energy=6 MeV and $\text{the field size}=20\times 20\;{\text{cm}}^{2}$.

*Calculation Parameters*	*3.2 GHZ Intel Xeon Processor with 2.8 GB of RAM (without DCF)*	*2.4 GHZ Intel Dual Quad core Xeon Processor with 24 GB of RAM (with DCF)*
$2\%,5\times 5\;{\text{mm}}^{2}$	1.08 min	0.23 min
2%, $1.5\times 1.5\;{\text{mm}}^{2}$	29.72 min	2.40 min
1%, $2\times 2\;{\text{mm}}^{2}$	68.20 min	3.57 min

In Table 4, the calculation time is higher for a better statistical accuracy and with a finer calculation grid size. Increasing the statistical precision or the calculation grid size necessitates an increase in the number of simulated particles and the calculation time is proportional to its number of particles. More specifically, the calculation time is inversely proportional to the square of the statistical accuracy. In Table 5, calculation time increases with the energy and the field size when using fixed statistical accuracy and grid size. To achieve the desired accuracy, higher energies necessitate a longer tracking of simulated particles, whereas increasing the field size requires more simulated particles. Thus, for both of these effects, the calculation time is increased. This is in agreement with the work of Neuenschwander et al.^(5)^ In Table 6, calculation times are really fast when using a parallelized dose calculation.

### B. Water phantom evaluation

**Figure 5:**
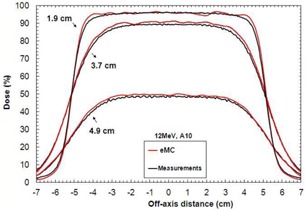
Measured and calculated crossbeam profiles in water at different depths (1.9 cm, 3.7 cm, and 4.9 cm) for a $10\times 10\;{\text{cm}}^{2}$ field size at 12 MeV and SSD=100 cm. Measurements were performed with an IC10 ionization chamber.

Table 7 shows depth‐dose curves analysis for three energies (6, 12, and 18 MeV) and three field sizes (6×6,10×10, and $20\times 20\;{\text{cm}}^{2}$) in water at an SSD of 100 cm. Figure 5 shows a comparison of measured and calculated profiles at 12 MeV for a 10×10 applicator, at SSD=100 cm. 6, 7 show profiles comparison of a 12 MeV beam with a $10\times 10\;{\text{cm}}^{2}$ field, respectively, for an extended 110 cm SSD and an obliquely incidence of 20°, at SSD=100 cm. Table 8 compares penumbras and maximum doses of beam profiles, at SSD=100 cm, at three different energies (6, 9, and 12 MeV), for a $10\times 10\;{\text{cm}}^{2}$ field.

**Figure 6:**
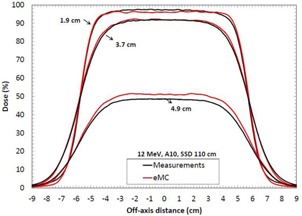
Measured and calculated crossbeam profiles in water at different depths (1.9 cm, 3.7 cm, and 4.9 cm) for a $10\times 10\;{\text{cm}}^{2}$ field size at 12 MeV and SSD=110 cm. Measurements were performed with an ionization chamber. All beam profiles were normalized according to the measured and calculated depth‐dose curve.

**Figure 7:**
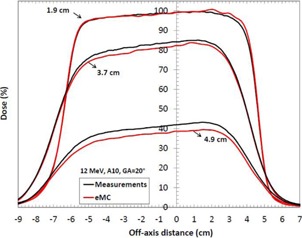
Measured and calculated crossbeam profiles in water at different depths (1.9 cm,3.7 cm, and 4.9 cm) for a $10\times 10\;{\text{cm}}^{2}$ field size, at 12 MeV and SSD=100 cm. Gantry angles $\text{GA}=20^{\circ }$. Measurements were performed with an ionization chamber. All beam profiles were normalized according to the measured and calculated depth‐dose curve.

**Table 7:** Maximum deviation (Δmax) between measured and calculated depth‐dose curves for different combinations of energy/applicator in water phantom at SSD=100 cm. The Δmax evaluation is separated into four groups: from 0.5 cm to depth to dmax, from dmax to depth of 80%, from depth of 80% to depth of 20%, and from depth of 20% to the end. Deviations are given in term of % dose difference, except in the gradient region (80%‐20%), where it is represented in term of distance to agreement (mm).

*Energy (MeV)*	*Field Size (cm^2^)*	Δ *max*			
		$0.5\;cm-d_{max}$	$d_{max}$–80%	*80%–20%*	*20%–end*
6	6×6	2.0%	2.0%	0.5 mm	2.0%
	10×10	2.0%	2.0%	0.3 mm	2.0%
	20×20	2.5%	2.5%	0.5 mm	2.0%
12	6×6	1.0%	2.0%	1.0 mm	2.5%
	10×10	0.3%	2.5%	1.0 mm	1.0%
	20×20	1.0%	2.0%	0.5 mm	1.5%
18	6×6	1.5%	1.5%	0.5 mm	0.3%
	10×10	2.0%	1.5%	1.0 mm	0%
	20×20	2.0%	1.0%	0.5 mm	0.3%

**Table 8:** Comparison of measured and calculated penumbras and dose of beam profiles in water at different depths, for three energies and for a field size of $10\times 10\;{\text{cm}}^{2}$ and a SSD of 100 cm. The symbol Δ represents the difference in cm between the measured and calculated penumbra, while the symbol $\Delta_{\text{max}}$ Dose represents the maximum difference of dose in % between the measured and the calculated beam profiles.

		*Penumbra*			
*Energy (MeV)*	*Depth (cm)*	*Measured (cm)*	*Calculated (cm)*	Δ (cm)	$\Delta_{max}$Dose (%)
6	0.8	0.65	0.74	0.09	1.0
	1.9	1.40	1.24	0.16	4.0
	2.2	1.40	1.36	0.04	3.5
12	1.9	0.78	0.79	0.01	1.5
	3.7	1.70	1.79	0.09	2.0
	4.9	2.09	2.06	0.03	1.4
18	2.5	0.83	0.81	0.02	1.6
	6.5	2.65	2.54	0.11	3.0
	7.3	2.84	2.68	0.16	3.2

In Table 7, maximum deviations of depth‐dose curves are all inside 2.5%, 1 mm, which respect Van Dyk's criteria.^(9)^ In 5, 6, 7, calculated beam profiles present oscillations due to the Monte Carlo's stochastic nature, but they are all in agreement with the measurements. Indeed, maximum deviations are all within 2%, except for Fig. 7, at depth of 4.9 cm, where the deviation is of 3.5%. However, this deviation corresponds only to a difference of 1.0 mm because it is in a large dose gradient region. In Table 8, penumbra differences are all within 1.6 mm, which is good. All maximum dose differences are inside 2% for depths near maximum dose. Also, differences are all inside 2 mm in the gradient region. Indeed, at 6 MeV, the gap of 4% in the gradient region corresponds to only a difference of 0.6 mm. At 12 MeV, the gap of 2% corresponds to a difference of 0.5 mm. Finally, at 18 MeV, the gap of 3% corresponds to a difference of 1.5 mm. All results for the water phantom evaluation meet or exceed the recommended limits set by Van Dyk et al.,^(9)^ regardless of the SSD or the gantry angle.

### C. Air cylinder phantom evaluation

**Figure 8:**
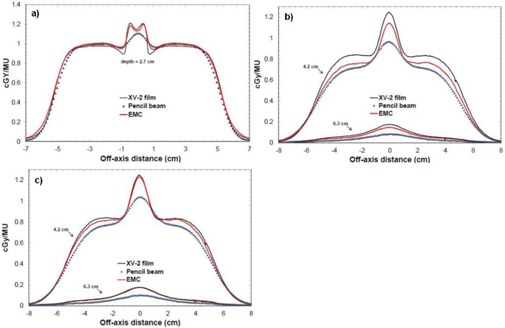
Crossbeam profiles in the air cylinder phantom for a $10\times 10\;{\text{cm}}^{2}$ applicator at 12 MeV and SSD=100 cm: (a) profile at the air cylinder–plastic water interface (2.7 cm of depth); (b) profiles in plastic water at two different depths (4.2 cm and 6.3 cm); (c) PWDT‐corrected profiles in plastic water (4.2 cm and 6.3 cm).

Figure 8 shows a comparison of beam profiles in the air cylinder phantom for a 12 MeV beam with a $10\times 10\;{\text{cm}}^{2}$ applicator. XV‐2 film measurements, pencil beam, and eMC calculations are presented. The first profile is at the air cylinder‐PWDT interface and the two others are in the PWDT material. In Fig. 8(a), the eMC calculation is in good agreement with the measurement for this depth, which corresponds approximately to the depth of maximum dose. However, in Fig. 8(b), there is a systematic difference between eMC calculation and the film measurement. This suggests a percentage depth‐dose difference between calculation and measurement. An analysis was performed to see if this difference came from the PWDT itself, which should be equivalent to water, according to technical specifications. The conclusion was that there is a difference between water and PWDT depth‐dose curves in the high gradient region. It means PWDT is not equivalent to water. The difference originates from a weakness in the algorithm during the geometric preprocessing, at the assignation of one of the five preset materials. In this case, PWDT is assigned to water material due to its similarity in their physical density. A material correction must be applied to the measurements in order to compensate the nonequivalence of PWDT to water. This is performed by multiplying a correction factor to all the beam profiles in the high gradient region (Fig. 8(b)). This factor varies with depth and it is obtained according to the depth‐dose curves analysis (PWDT versus water). Figure 8(c) compares the film measurements in plastic water (beam profiles of Fig. 8(b)) with corrected calculated doses for both algorithms.

### D. Lung inhomogeneity phantom evaluation

**Figure 9:**
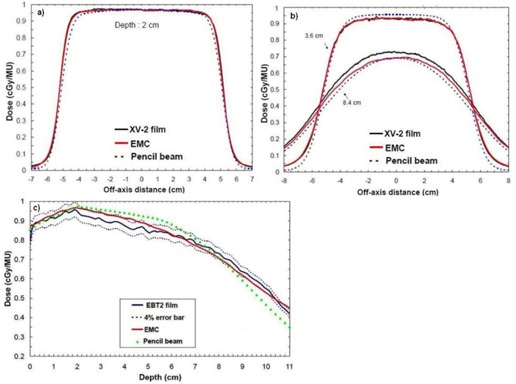
Crossbeam profiles in the first version of the lung phantom and a depth‐dose curve in the second version of the lung phantom (12 MeV, $10\times 10\;{\text{cm}}^{2}$, SSD=100 cm): (a) profile at the plastic water–cork interface (2 cm of depth); (b) profiles in the cork at different depths (3.6 cm and 8.4 cm); (c) depth‐dose curve (0–2 cm water, 2–11 cm cork).

Figures 9(a) and 9(b) show a comparison of beam profiles in the first version of the lung phantom for a 12 MeV beam with a $10\times 10\;{\text{cm}}^{2}$ applicator. Here, film measurements, eMC and pencil beam calculations are presented. The first profile is at the plastic water–cork interface and the two others are in the cork. In both figures, beam profiles results show good agreement between eMC and measurements. The maximum observed discrepancy is 4%.

Figure 9(c) shows a comparison of percentage depth‐dose curves in the second version of the lung phantom for a 12 MeV beam with a $10\times 10\;{\text{cm}}^{2}$ applicator. Depth‐dose curve results show differences between both algorithms at the beginning of the water–cork interface. The eMC depth dose is inside the 4% error bar of the EBT2 film depth dose. The pencil beam algorithm predicts incorrect doses in the cork part.

### E. Chest wall phantom evaluation

**Figure 10:**
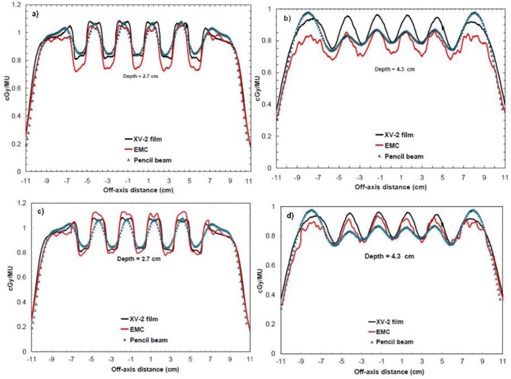
Crossbeam profiles in the chest wall phantom for a $20\times 20\;{\text{cm}}^{2}$ applicator at 12 MeV and SSD=100 cm: (a) profile at 2.7 cm of depth; (b) profile at 4.3 cm of depth; (c) profile at 2.7 cm of depth, with the stopping power correction for the Teflon part; (d) profile at 4.3 cm of depth, with the stopping power correction for the Teflon part.

Figures 10(a) and 10(b) show a comparison of beam profiles at different depths in the chest wall phantom for a 12 MeV beam with a $20\times 20\;{\text{cm}}^{2}$ applicator. Here once again, film measurements, pencil beam, and eMC calculations are presented. Relatively, the eMC algorithm is in good agreement with the film measurements. However, in absolute it is not as good. Beam profiles are 5% off at 2.7 cm of depth and 10% off at 4.3 cm of depth. Considering how the algorithm assigns a voxel of a patient computed tomography (CT) image volume to one of the five preset materials in the database (air, lung, water, Lucite, and bone) based on the mass density (given by the CT number), differences shown in Figs. 10(a) and 10(b) are due to an inappropriate material assignation. In fact, the Teflon material of $2.16\;g/{\text{cm}}^{3}$ of density is automatically assigned to the bone material (SB3) of $1.84\;g/{\text{cm}}^{3}$ of density, which is the highest density preset material. The SB3 material has a Zeff of 13.9, while the one for Teflon is of 8.4, meaning that the stopping power is higher for the SB3 material and this falsifies the calculated dose distributions. With the bone material composition being known,^(14)^ it is possible to calculate the total corresponding stopping powers and to compare it with Teflon. From the NIST Web site,^(15)^ the obtained ratio of total stopping power of the SB3 material over the Teflon material, on the 0–20 MeV energy range, is 1.076 with a small standard deviation of 0.027 — meaning that electrons deposit on average 7.6% more dose in the SB3 material than in the Teflon material. Figures 10(c) and 10(d) show beam profiles that include the stopping power correction only for the Teflon region in the chest wall phantom. More specifically, Teflon dose voxels were multiplied by 7.6% for every beam profile at different depths (2.7 cm and 4.3 cm). Corrected, eMC beam profiles now coincide with the measurements.

### F. RANDO phantom evaluation

**Figure 11:**
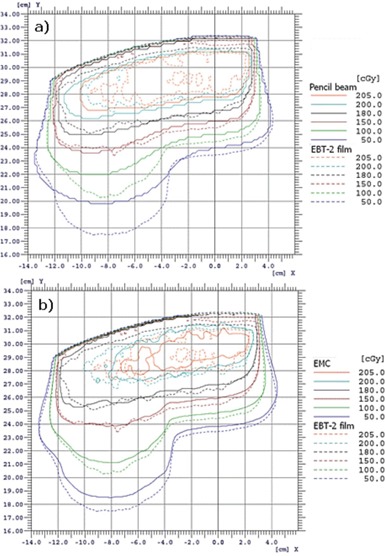
Illustration of superposed measured and calculated absolute isodoses in RANDO for a $15\times 15\;{\text{cm}}^{2}$ applicator at 18 MeV and SSD=100 cm: (a) pencil beam algorithm vs. EBT2 film; (b) eMC algorithm vs. EBT2 film.

Figure 11(a) shows the superposition of absolute isodoses calculated with the pencil beam algorithm and measured with a EBT2 film in RANDO, whereas Fig. 11(b) shows the superposition of absolute isodoses calculated with the eMC algorithm and measured with a EBT2 film.

In Fig. 11(a), the pencil beam algorithm does not deposit enough doses with depth in RANDO's lung part, comparatively to measurement. There are up to 2 cm of differences with the measurement. In Fig. 11(b), the eMC calculation is in good agreement with the measurement. There is a small difference (<0.8 cm) for the 50 cGy and the 100 cGy isodoses, which are high‐dose gradient doses. This 8 mm offset in the lung corresponds to a 2 mm offset in water, and this meets the criteria of acceptability set by Van Dyk et al.^(9)^

### G. Clinical case data

**Figure 12:**
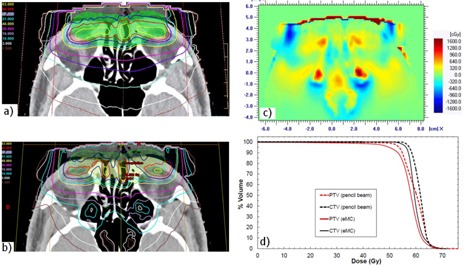
Illustration (a) of the absolute isodoses from the pencil beam algorithm on a CT slice of the clinical case (doses in Gy: 1.5, 3, 10, 18, 30, 40, 51, 57, 60, 63). Illustration of the absolute isodoses from the eMC algorithm (b) on the same CT slice of (a). The planned target (PTV) volume is defined by the green shadowed region and the clinical target volume (CTV) is defined by the orange shadowed region. In (a) and (b), the prescription dose is 60 Gy and 210 MU were delivered for both algorithms. Map (c) of dose differences as calculated by the two planning systems (pencil beam minus eMC). DVH graph (d) for the PTV and the CTV for both algorithms.

Figure 12(a) presents absolute isodoses obtained from the pencil beam algorithm, while Fig. 12(b) presents those which are obtained from the eMC algorithm. For both algorithms, 210 MU were delivered and the prescription dose is 60 Gy. The planned target volume (PTV) is defined by the green shadowed region near the surface and the clinical target volume (CTV) is defined by the orange shadowed region. Figure 12(c) presents a difference map of planar dose between pencil beam and eMC algorithms. Figure 12(d) is a DVH graph of the PTV and the CTV for both algorithms.

In Fig. 12(c), the difference map of planar dose between the pencil beam and the eMC algorithm shows large differences, ranging up to 20% in the sinus. The eMC calculations more precisely predict large dose perturbations due to inhomogeneities. In Fig. 12(d), the pencil beam algorithm predicts higher doses for the CTV and the PTV when compared to the eMC algorithm. The CTV and PTV volumes covered by the prescription dose are, respectively, 34% and 25% higher for the pencil beam algorithm.

## IV. DISCUSSION

As demonstrated in Fig. 3, Table 1, and Table 2, no matter which seed number is used, the calculation respects the accuracy entered by the planner (in our case 2%, 2 mm). Therefore, it is permitted to use the same seed number for all calculations. In Fig. 4, it was shown that, for a 6 MeV electron beam, a $1\times 1\;{\text{mm}}^{2}$ grid size gives a better result when compared to a $2\times 2\;{\text{mm}}^{2}$ grid size, which coincide with Popple et al.^(6)^ criterion. The calculation of 1% of resolution does not significantly improve the calculations compared with the 2%, and it also takes longer to calculate. A calculation curve with a 2% of resolution with no filter being applied will vary a little bit more than a calculation curve having a 1% resolution; however, when a smooth median 2D filter is applied (which is the case), the curves are similar. This study was also performed with other energies and conclusions are similar to that of the Popple study. The important thing is to ensure the calculation grid is small enough for the actual energy chosen. A 1% resolution versus 2% has no major impact. There is a discrepancy at shallow depth of Fig. 4, which does not exist in Fig. 3. It can be explained by the fact that, in Fig. 3, the percentage depth‐dose curves are for high energy, 18 MeV, while Fig. 4 presents percentage depth‐dose curves for low energy, 6 MeV. The hypothesis is that, at low energy, the kugel size is too large, combined with the approximation of deposit of rectilinear dose between the entrance and the exit of the kugel, resulting in the model being unable to simulate the fast increase of the angular dispersion at the entrance of the phantom. This problem does not exist at higher energy. As seen in Table 3, the output factor agreements were generally within 2%, except for cutouts of $3\times 3\;{\text{cm}}^{2}$ where the agreement was within 4%. This can be explained by both the measurement and the eMC uncertainties. Compared to calculation times of 4, 5, those from Table 6 are really much faster because of the use of parallelized dose calculations. Under these conditions, the eMC algorithm can be considered for routine treatment planning. For the water phantom evaluation, calculated depth‐dose curves and beam profiles at different SSDs (100 cm and 110 cm) and gantry angles $\left(\text{GA}=0^{\circ }\;\text{and}\;G=20^{\circ }\right)$ met the recommended Van Dyk criteria.^(9)^ For the air cylinder phantom evaluation, the observed difference in Fig. 8(b) originates from a weakness of the algorithm during the geometric preprocessing, at the assignation of one of the five preset materials. In this case, PWDT is assigned to water material due to the similarity of their physical density. A material compensation must be applied to measurements in order to correct the nonequivalence of PWDT to water. In Fig. 8(c), the eMC calculations are now in good agreement with the measurements. The PBA algorithm does not predict details near the interface of the inhomogeneity. At 4.2 cm, there is an under dosage of 15% under the air cylinder and another of 10% around the cylinder. At 6.3 cm, discrepancies are even still higher. The PBA algorithm does not predict the large dose perturbations due to small low‐density inhomogeneities. As seen in Fig. 9, beam profiles and depth‐dose curves for the lung inhomogeneity phantom are in good agreement with the eMC and the measurements. The PBA algorithm is not as optimal. It incorrectly predicts higher doses in the cork part. This suggests that PBA does not model realistically electron scattering in low‐density tissues. Beam profiles in Figs. 10(c) and 10(d), which were corrected for the Teflon material stopping power in the chest wall phantom, are in good agreement with the film measurements. Compared to the PBA, the eMC calculations more precisely predict large dose perturbations due to inhomogeneities. The material assignation method of the eMC algorithm is not a problem for clinical cases where the materials in question are actual soft tissues and real bones; they are similar to the five preset materials which are representative of human tissues encountered in a clinical environment. Finally, both the anthropomorphic phantom (thorax part) evaluation and the clinical case (nose) evaluation show large differences between the pencil beam algorithm and the eMC algorithm in low‐density regions. The eMC algorithm models realistically dose perturbations in those two phantoms. For the nose case presented in Fig. 12, the PBA algorithm shows a better target coverage as compared to the eMC algorithm; however, it is not realistic. When looking at the eMC calculated DVHs in Fig. 12, we can see the number of MU calculated by the PBA algorithm and delivered to the patient should have been increased to obtain the desired coverage of the CTV and PTV. Also, the overdosage and underdosage zones of the eMC calculation localized around the PTV could affect normal structures, those being the optical system and lachrymal glands. This clinical aspect is more important for reirradiation, where dose tolerances for organs at risk are more critical. Thus, for the clinical case presented in Fig. 12, the eMC algorithm provides a more realistic illustration of the dose distribution with its hot and cold spots. The eMC algorithm is a better tool for the radiation oncologist to evaluate the treatment plan and take the appropriate clinical decisions.

## V. CONCLUSIONS

In this study, the eMC algorithm showed good agreements with the measurements in simple homogeneous and heterogeneous phantoms. Compared to the electron pencil beam algorithms, the eMC calculations predicted more accurately large dose perturbations due to inhomogeneities. The eMC algorithm can be considered for routine treatment planning. In particular, the parallel computing feature enables fast calculation compatible with efficient clinical workflow.
